# Association Between Vitamin D Receptor rs731236 (Taq1) Polymorphism and Risk for Restless Legs Syndrome in the Spanish Caucasian Population

**DOI:** 10.1097/MD.0000000000002125

**Published:** 2015-10-30

**Authors:** Félix Javier Jiménez-Jiménez, Elena García-Martín, Hortensia Alonso-Navarro, Carmen Martínez, Martín Zurdo, Laura Turpín-Fenoll, Jorge Millán-Pascual, Teresa Adeva-Bartolomé, Esther Cubo, Francisco Navacerrada, Ana Rojo-Sebastián, Lluisa Rubio, Sara Ortega-Cubero, Pau Pastor, Marisol Calleja, José Francisco Plaza-Nieto, Belén Pilo-De-La-Fuente, Margarita Arroyo-Solera, Esteban García-Albea, José A.G. Agúndez

**Affiliations:** From the Section of Neurology, Hospital Universitario del Sureste, Arganda del Rey (FJJ-J, HA-N, FN, MC, JFP-N, BP-D-LF, MA-S); Department of Pharmacology, University of Extremadura, Cáceres; Department of Medicine-Neurology, Hospital “Príncipe de Asturias”, Universidad de Alcalá, Alcalá de Henares, Madrid (AR-S, LR, EG-A, FJJ-J, HA-N); Department of Pharmacology, Universidad de Extremadura, Cáceres (EG-M, JAGA); Department of Pharmacology, University of Extremadura, Badajoz (CM); Section of Neurology, Hospital Virgen del Puerto, Plasencia, Cáceres (MZ); Section of Neurology, Hospital La Mancha-Centro, Alcázar de San Juan, Ciudad Real (LT-F, JM-P); Unit of Neurology, Clínica Recoletas, Zamora (TA-B); Section of Neurology, Hospital Universitario de Burgos, Burgos (EC); CIBERNED,Centro de Investigación Biomédica en Red de Enfermedades Neurodegenerativas, Instituto de Salud Carlos III (SO-C, PP); Neurogenetics Laboratory, Division of Neurosciences, Center for Applied Medical Research, Universidad de Navarra, Pamplona (SO-C, PP); Department of Neurology, Clínica Universidad de Navarra, University of Navarra School of Medicine, Pamplona (SO-C, PP); and Department of Neurology, Hospital Universitari Mutua de Terrassa, Terrassa, Barcelona, Spain (PP).

## Abstract

Several recent works suggest a possible role of vitamin D deficiency in the etiology or restless legs syndrome (RLS). We analyzed the possible relationship of 2 common single nucleotide polymorphisms (SNPs) in the *vitamin D3 receptor* (*VDR*) gene with the risk for RLS.

We studied the genotype and allelic variant frequencies of *VDR rs2228570* and *VDR rs731236* SNPs in 205 RLS patients and 445 healthy controls using a TaqMan essay.

The frequencies of the rs731236AA genotype and the allelic variant rs731236A were significantly lower in RLS patients than in controls (*P* < 0.005 and < 0.01, respectively). Restless legs syndrome patients carrying the allelic variant rs731236G had an earlier age at onset, and those carrying the rs731236GG genotype had higher severity of RLS, although these data disappeared after multivariate analyses. None of the SNPs studied was related with the positivity of family history of RLS.

These results suggest a modest, but significant association between *VDR rs731236* SNP and the risk for RLS.

## INTRODUCTION

Genetic factors are likely to be very important in the etiology of restless legs syndrome (RLS or Willis-Ekbom disease—WED), but all the responsible gene(s) remain(s) to be identified (revised in reference).^1^ Genome Wide Association Studies (showed association between the risk for RLS and variants of several genes, including Protein Tyrosine Phosphatase Receptor Type Delta (PTPRD, chromosome 9p24.1–p23) BTB/POZ Domain Containing Protein 9 (BTBD9, chromosome 6p21), MEIS1 (chromosome 2p14p13), mitogen-activated protein kinase 5/SKI family transcriptional corepressor 1 (MAP2K5/SKOR1, chromosome 15q23), and the variants rs6747972 at chromosome 2p14, and rs3104767 at chromosome 16q12.1.^1^ Whole exome sequencing studies found association or RLS risk with some variants in the protocadherin alpha 3 (PCDHA3, chromosome 5q31) in a German family.^1^

The pathophysiology of idiopathic RLS (iRLS) is not well understood as well. Whereas iron deficiency and dopaminergic dysfunction are the main pathogenic hypothesis, recent works suggest the possible implication of neurotransmitters and/or neuromodulators such as aspartate, glutamate, opiates, or gamma-hydroxybutyric acid (GABA) (revised in reference).^2^ Moreover, in the last years, several interesting reports pointed out a possible role of *vitamin D* deficiency (which eventually could cause alterations in the development of the dopaminergic system),^3,4^ in the etiology of iRLS:

1. A case-control study found decreased serum 25-hydroxyvitamin D in female with iRLS, which was inversely correlated with disease severity.^5^
2. Increased prevalence of RLS described in patients with musculoskeletal symptoms with relatively lower when compared with those patients of the same cohort with higher serum 25-dihydroxyvitamin levels.^6^
3. Increased levels of vitamin D-binding protein in the CSF of RLS patients found in a preliminary case-control study using proteomic analysis of the CSF.^7^
4. Improvement in the severity of RLS symptoms with administration of vitamin D supplements to patients with vitamin D deficiency found in an open-label study involving 12 patients.^8^

*Vitamin D3* (1,25-dihydroxyvitamin D3) *receptor* gene (*VDR*, *NR1I1* or *PPP1R163*; chromosome 12q13.11; Gene ID 7421; MIM 601769) encodes the nuclear hormone receptor for vitamin D3. This receptor has a similar sequence to the thyroid and steroid receptors, belongs to a family of trans-acting transcriptional regulatory factors and acts as a receptor for the lithocolic acid. It is involved in mineral metabolism and other metabolic pathways involved in cancer and in the immune response (link http://www.ncbi.nlm.nih.gov/gene/7421).

*VDR* gene shows 2 common single nucleotide polymorphisms (SNP) in Caucasians: the SNP rs2228570 (*Fok1*), which causes the amino acid substitution (Met 1 Thr), and the synonymous SNP rs731236 (*Taq1*), which does not cause amino acid substitution (Ile 352 Ile), but it has been studied with regard to several clinical conditions. These SNPs have been associated with the risk for multiple sclerosis by several studies, although a meta-analysis of these studies did not confirm such association.^9^ The rs731236 and other 2 SNPs in the *VDR* gene showed lack of association with Parkinson's disease in a meta-analysis study.^10^

Despite VDR polymorphisms have not been mentioned among the possible susceptibility genes for RLS in GWAS, it seems to be reasonable, due the possible role of vitamin D deficiency in the pathogenesis of RLS, to study the association between SNPs related with vitamin D and the risk for RLS. For this purpose, we genotyped rs2228570 (*Fok1*) and rs731236 (*Taq1*) in the *VDR* gene in Caucasian Spanish RLS patients and controls.

## PATIENTS AND METHODS

### Patients and Controls

Two-hundred and five unrelated patients diagnosed with iRLS according with established RLS diagnostic criteria,^11,12^ and 445 gender-matched healthy controls were included in the study (Table 1). Consultant neurologists with expertise in Movement Disorders recruited the RLS patients. Besides the diagnosis of iRLS, the absence of previous neurological or systemic diseases, and the exclusion of possible causes of secondary RLS such as peripheral neuropathy, renal failure, anaemia, rheumatoid arthritis, and exposure to drugs able to induce or to aggravate RLS, were required previously to the inclusion of patients in the study. For this purpose, RLS patients underwent laboratory studies (blood count, routine biochemistry, serum levels of vitamin B_12_, folic acid, and thyroid hormones, iron metabolism studies, proteinogram, rheumatoid factor, antinuclear antibodies, and nerve conduction studies). Restless legs syndrome severity was assessed by using the International RLS Study Group Rating Scale (IRLSSGRS).^13^

**TABLE 1:**
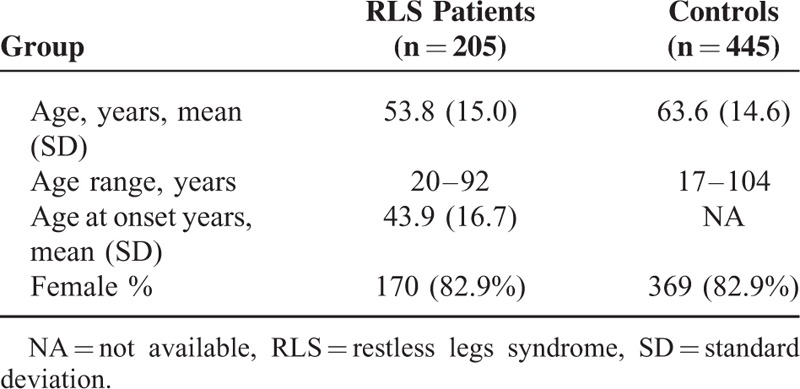
Demographic Data of the Series Studied

The 445 controls were healthy Caucasian Spanish individuals (none of them having RLS, tremor or other movement disorders, or systemic diseases) matched by gender (275 of them were recruited from the Infanta Cristina University Hospital, Badajoz, Spain; and the remaining 270 were recruited at the Clínica Universitaria de Navarra; Pamplona, Spain).

### Ethical Aspects

All the participants were included in the study after giving their written informed consent when the procedure of the study was full explained. The Ethics Committees of Clinical Investigation of the University Hospital “Príncipe de Asturias” (University of Alcalá, Alcalá de Henares, Madrid, Spain), the Clínica Universitaria de Navarra (Pamplona, Spain), the Infanta Cristina University Hospital (Badajoz, Spain), and the Province of Cáceres (Cáceres, Spain), approved the study, that was conducted according with the principles enumerated in the Helsinki Declaration of 1975. Most of the patients recruited had participated in other previous case-control studies of genetic association with the risk for RLS.^14–18^

### Genotyping of VDR Polymorphisms

We studied 2 SNPs in the *VDR* gene. The rs2228570 SNP causes the amino acid substitution Met 1 Thr and that designated as rs731236 is a synonymous Ile 352 Ile SNP. The former SNP was selected because it is the only nonsynonymous VDR SNP that shows a frequency >0.01 in Caucasian individuals. The later SNP was selected because it is the only synonymous VDR SNP that has a high frequency (∼0.40 in Caucasians) and because it has several clinical associations. These 2 variants do not show linkage between themselves (D′ = 0.06; *r*^2^ = 0.003) according the 1000 genomes integrated phase 1.

Genomic DNA was obtained from peripheral leukocytes and purified according to standard procedures. *VDR* genotyping was carried out by means of custom TaqMan Assay (Applied Biosciences Hispania, Alcobendas, Madrid, Spain) designed to detect the SNPs rs2228570 (C_12060045_20) and rs731236 (C_2404008_10). The full procedure was reported elsewhere.^9^

### Statistical Analysis

We used the DeFinetti program (http://ihg.gsf.de/cgi-bin/hw/hwa1.pl), to assess the Hardy–Weinberg equilibrium, the PLINK software^19^ to perform the allele and genotype analysis, and the PHASE v2.1.1 program^20^ to perform the haplotype reconstruction, using a default model for recombination rate variation with 1000 iterations, 500 burn-in iterations, and a thinning interval of 1. The combination of haplotypes in the best run (the one that showed the maximum consistency of results across all runs) was used to obtain diplotypes.^21^

Statistical analyses were performed using the SPSS 19.0 for Windows (SPSS Inc, Chicago, IL). The χ2 or Fisher tests were used to calculate the intergroup comparison values when appropriate, and the 95% confidence intervals were calculated as well. Multiple comparison of means was done by using the Kruskal–Wallis test for independent samples, and correction for multiple testing (Pc values) was done with the False Discovery Rate procedure.^22^

The determination of the sample size was done from variant allele frequencies observed in control individuals with a genetic model analyzing the frequency for carriers of the disease gene with an RR value = 1.5 (*P* = 0.05). The statistical power for 2-tailed associations for the presence of the SNPs identified in this study (rs2228570 and rs731236) was 81.9%, and 82.6%. The Breslow–Day test was used to perform analysis for heterogeneous genetic association (homogeneity test).

We calculated the negative predictive value (NPV) as *d*/*r*^2^ ratio (*d* = number of control individuals with the risk factor absent; *r*^2^ = sum of patients and controls with the risk factor absent).^23^

## RESULTS

The frequencies of the *rs2228570* and r*s731236* genotypes and allelic variants were in Hardy–Weinberg's equilibrium, both in RLS patient and control groups (Table 2). The frequencies of *rs731236AA* genotype and *rs731236A* allele were significantly lower in RLS patients than in controls, both in the whole series (Table 2) and in female gender (Table 3), and remained significant after multiple comparison analysis according the false discovery rate correction. Armitage's test for trend revealed a gene-dose effect for the whole series (chi square = 7.09; *P* = 0.00773), and for women (chi square = 9.34; *P* = 0.00224), which suggest an incomplete penetrance. The Breslow–Day test indicates that the association was homogeneous (*P* = 0.284 and *P* = 378), for the whole series, and for women, respectively. Restless legs syndrome patients carrying the allelic variant *rs731236G* had an earlier onset of RLS symptoms (Table 4), and RLS patients with *rs731236GG* genotype showed higher severity of RLS symptoms (Table 4). The frequencies of the *rs2228570* genotypes and allelic variants did not differ significantly between RLS patients and controls (Table 2), were not influence by gender (Table 3), and were unrelated both with the age at onset of RLS (Table 4) and with the severity of RLS symptoms (Table 4). The distribution of genotypes and allelic frequencies of the 2 SNPs studied was similar in RLS patients with positive family history of RLS than in those with negative family history of RLS (Table 5).

**TABLE 2:**
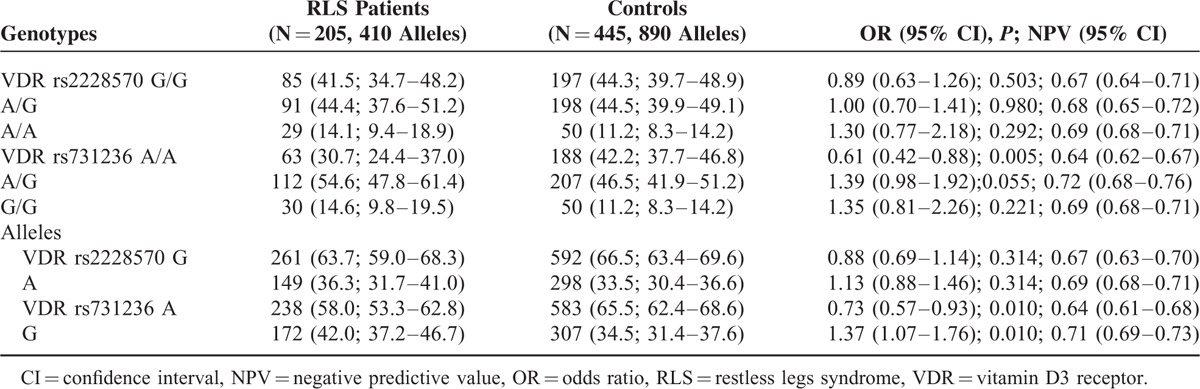
VDR Genotypes and Allelic Variants of Patients With RLS and Healthy Volunteers. The Values in Each Cell Represent: Number (Percentage; 95% Confidence Intervals)

**TABLE 3:**
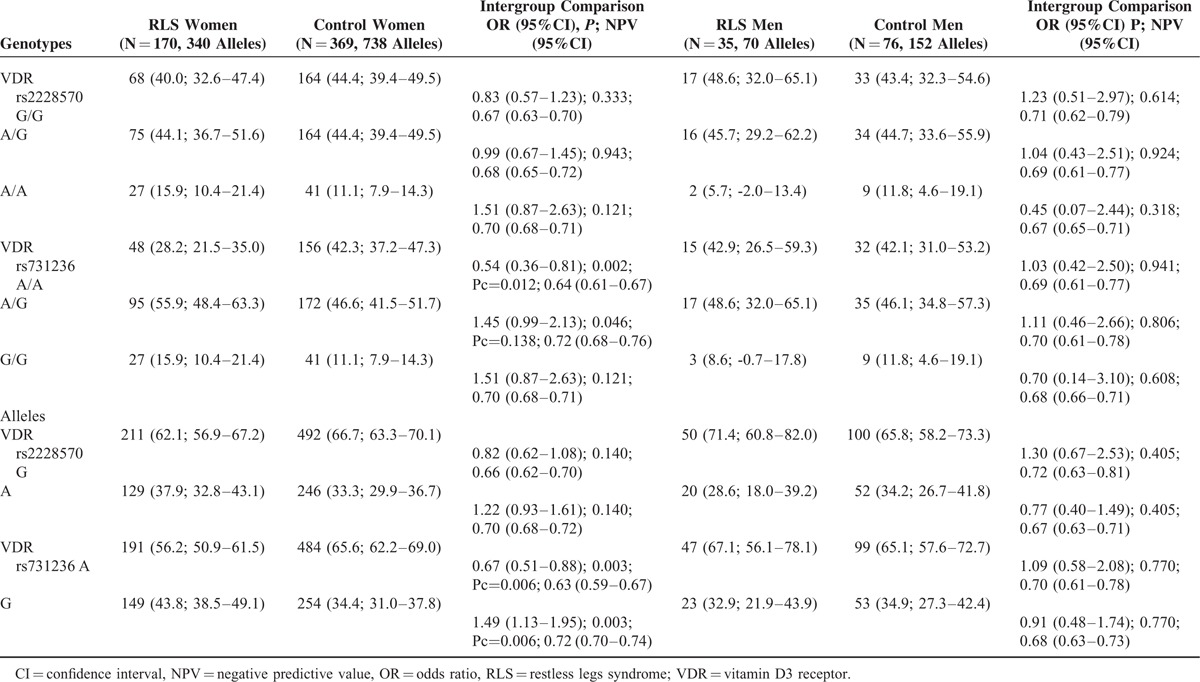
VDR rs2228570 and VDR rs731236 Genotypes and Allelic Variants of Patients With RLS and Healthy Volunteers Distributed by Gender. The Values in Each Cell Represent: Number (Percentage; 95% Confidence Intervals). Pc Probability After Correction for Multiple Comparisons

**TABLE 4:**
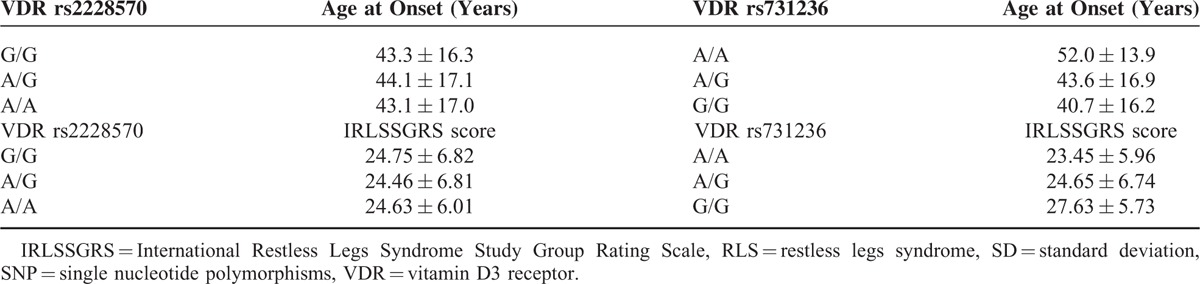
Mean + SD Age at Onset (Years) of RLS Symptoms and Mean + SD IRLSSGRS Score According With the VDR Genotypes. Both Age at Onset and IRLSSGRS Score Were Dependent of the SNP rs731236 (Kruskal–Wallis Test for Independent Samples *P* = 0.009 Each)

**TABLE 5:**
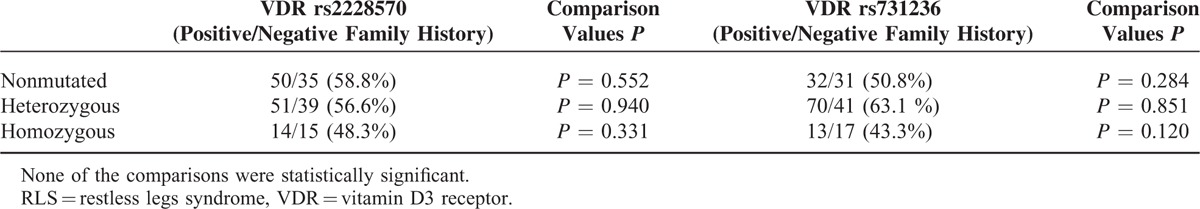
VDR Genotypes According to Family History or RLS

Linear regression under the standard additive model, which included in a single model the genotypes, gender, age, age at onset, ferropenia, and IRLSSGRS, was performed. Aside from the association of the SNP rs731236 with RLS risk, none of the putative associations were statistically significant.

## DISCUSSION

Familial RLS shows usually an autosomal dominant inheritance pattern (less frequently, nonmendelian or autosomal recessive patterns have been described). To date, at least 8 genes/loci have been identified in linkage studies. Association between several variants of the *PTPRD, MEIS1*, *BTBD9*, and *MAP2K5*/*SKOR1* genes with the risk of developing RLS have been found in GWAS, and between the *PCDHA3* gene and the risk for RLS in an exome sequencing study (revised in^1^). The results of the few case-control association studies reported in RLS are inconclusive (revised in^1^). Previous case-control association studies reported by our group involving the same cohort of the present study showed a weak association between *heme-oxygenase 1 (HMOX1)* rs2071746 polymorphism and the risk to develop RLS,^18^ whereas no association was found with several SNPs in the *microtubule-associated protein tau (MAPT)*,^14^*dopamine receptor D3 (DRD3)*,^15^*solute carrier family 1-(glial high affinity glutamate transporter-), member 2 (SLC1A2),*^16^ and *nitric oxide synthase 1 (neuronal) (NOS1* or *nNOS)* genes.^17^

Vitamin D has several important effects on the dopaminergic system. Developmental vitamin D deficiency in rats induces increase in dopamine content in the cortex and hypothalamus, and increase in dihydroxyphenylacetic (DOPAC) acid (one of the main metabolites of dopamine) and noradrenalin levels in the cortex,^24^ increase in dopamine transporter density in the striatum and of affinity in the nucleus accumbens.^25^

A recent experimental study in rats showed that chronic administration of 1,25-dihydroxyvitamin D induces changes in several neurotransmitters, which included increase in tyrosine-hydroxylase (the rate-limiting enzyme in the synthesis of dopamine) and tryptophan hydroxylase 2 expression, increased concentrations of dopamine and serotonin metabolites, and increase in monoamine oxidase A (MAO-A) expression in the brain.^26^ These changes can cause alterations in the homeostasis of dopaminergic (of high importance in the pathogenesis of RLS) and serotonergic neurotransmission. Interestingly, VDR is widely expressed in the human brain, including the striatum and the nucleus accumbens.^27^

*VDR s2228570* is a 5′ variant which is located between the blocks 2 and 3 of the *VDR* gene, its functional consequence is that T > C eliminates translation start site, and the affected biological processes and phenotypes include calcium absorption, calcium accretion to skeleton and bone mineral density at different ages, vitamin D and parathyroid hormone levels, calcipotriol response in psoriasis, effects on antimycobacterial therapy, cell viability in thyroid cancer, and growth inhibition in breast cancer.^28^*VDR rs731236* is a 3′ variant located in the block 5 in the *VDR* gene, its functional consequence is T > C methylation site, and the affected biological processes and phenotypes include calcipotriol response in psoriasis, remission period of chronic plaque psoriasis treated with narrow band ultraviolet light, tuberculosis susceptibility, effects of vitamin D supplement in new fractures in postmenopausal women and in bone mineral density in adolescent girls.^28^

Data from the present case-control association study suggest a weak association of the *VDR rs731236*, but not of the *rs2228570* polymorphism, with the risk for RLS, together with an influence of that SNP in the age at onset and the severity of RLS. These findings are potentially interesting taking in account the possible relationship between vitamin D and RLS previously suggested by several preliminary reports.^5,8^

However, the results of the present study should be taken with caution because it has several limitations. These include the relatively low sample size, the lack of previous similar studies in other populations, and a selection bias with a relatively high male-to-female ratio in our RLS patients (likely due to the clinical setting of the patients’ recruitment). In addition, because data on the possible role of vitamin D in the pathogenesis of RLS are relatively recent, we did not measure serum vitamin D levels in our patients and controls cohorts at the time of enrollment to participate in genetic studies.

Taken in account the previously mentioned limitations, our results point to a modest association of the *VDR rs731236* polymorphism with the risk for RLS in Spanish Caucasian individuals and give a little support to the hypothesis of the relationship of vitamin D deficiency with the etiopathogenesis of RLS. Future studies combining the measurement of serum vitamin D levels with genotyping of *VDR* polymorphisms are warranted.

## References

[1] Jiménez-Jiménez FJ Alonso-Navarro H García-Martín E Latest perspectives in genetic risk factors for restless legs syndrome. *Eur Neurol Rev* 2013; 90–96.

[2] Jiménez-Jiménez FJ Alonso-Navarro H García-Martín E Neurochemistry of idiopathic restless legs syndrome. *Eur Neurol Rev* 2015; 10:35–44.

[3] Cui X Pelekanos M Burne TH Maternal vitamin D deficiency alters the expression of genes involved in dopamine specification in the developing rat mesencephalon. *Neurosci Lett* 2010; 486:220–223. 20884326 10.1016/j.neulet.2010.09.057

[4] Eyles DW Burne TH McGrath JJ Vitamin D, effects on brain development, adult brain function and the links between low levels of vitamin D and neuropsychiatric disease. *Front Neuroendocrinol* 2013; 34:47–64. 22796576 10.1016/j.yfrne.2012.07.001

[5] Balaban H Yıldız ÖK Çil G Serum 25-hydroxyvitamin D levels in restless legs syndrome patients. *Sleep Med* 2012; 13:953–957. 22704399 10.1016/j.sleep.2012.04.009

[6] Oran M Unsal C Albayrak Y Possible association between vitamin D deficiency and restless legs syndrome. *Neuropsych Dis Treat* 2014; 10:953–958. 10.2147/NDT.S63599 PMC4039397 24899811

[7] Patton SM Cho YW Clardy TW Proteomic analysis of the cerebrospinal fluid of patients with restless legs syndrome/Willis–Ekbom disease. *Fluids Barriers CNS* 2013; 10:20. 23758918 10.1186/2045-8118-10-20 PMC3680184

[8] Wali S Shukr A Boudal A The effect of vitamin D supplements on the severity of restless legs syndrome. *Sleep Breath* 2015; 19:579–583. 25148866 10.1007/s11325-014-1049-y

[9] García-Martín E Agúndez JA Martínez C Vitamin D3 receptor (VDR) gene rs2228570 (Fok1) and rs731236 (Taq1) variants are not associated with the risk for multiple sclerosis: results of a new study and a meta-analysis. *PLoS One* 2013; 8:e65487. 23840333 10.1371/journal.pone.0065487 PMC3688728

[10] Zhang ZT He YC Ma XJ Association between vitamin D receptor gene polymorphisms and susceptibility to Parkinson's disease: a meta-analysis. *Neurosci Lett* 2014; 578:122–127. 24993298 10.1016/j.neulet.2014.06.051

[11] Allen RP Picchietti D Hening WA Restless legs syndrome: diagnostic criteria, special considerations, and epidemiology: a report from the restless legs syndrome diagnosis and epidemiology work shop at the National Institute of Health. *Sleep Med* 2003; 4:101–119. 14592341 10.1016/s1389-9457(03)00010-8

[12] Allen RP Picchietti DL Garcia-Borreguero D International Restless Legs Syndrome Study Group 2014. Restless legs syndrome/Willis–Ekbom disease diagnostic criteria: updated International Restless Legs Syndrome Study Group (IRLSSG) consensus criteria—history, rationale, description, and significance. *Sleep Med* 2014; 15:860–873. 25023924 10.1016/j.sleep.2014.03.025

[13] Walters AS LeBrocq C Dhar A Validation of the International Restless Legs Syndrome Study Group rating scale for restless legs syndrome. *Sleep Med* 2003; 4:121–132. 14592342 10.1016/s1389-9457(02)00258-7

[14] Roco A Jiménez-Jiménez FJ Alonso-Navarro H MAPT1 gene rs1052553 variant is unrelated with the risk for restless legs syndrome. *J Neural Transm* 2013; 120:463–467. 23001634 10.1007/s00702-012-0897-5

[15] Jiménez-Jiménez FJ Alonso-Navarro H Martínez C Dopamine Receptor D3 (DRD3) gene rs6280 variant and risk for restless legs syndrome. *Sleep Med* 2013; 14:382–384. 10.1016/j.sleep.2012.11.009 23312624

[16] Jiménez-Jiménez FJ Alonso-Navarro H Martínez C The solute carrier family 1 (glial high affinity glutamate transporter), member 2 gene, SLC1A2, rs3794087 variant and assessment risk for restless legs syndrome. *Sleep Med* 2014; 15:266–268. 24424098 10.1016/j.sleep.2013.08.800

[17] Jiménez-Jiménez FJ Alonso-Navarro H Martínez C Neuronal nitric oxide synthase (nNOS, NOS1) rs693534 and rs7977109 variants and risk for restless legs syndrome. *J Neural Transm* 2015; 122:819–823. 25300364 10.1007/s00702-014-1322-z

[18] García-Martín E Jiménez-Jiménez FJ Alonso-Navarro H Heme oxygenase-1 and 2 common genetic variants and risk for restless legs syndrome. *Medicine (Baltimore)* 2015; 94:e1448. 26313808 10.1097/MD.0000000000001448 PMC4602895

[19] Purcell S Neale B Todd-Brown K PLINK: a tool set for whole-genome association and population-based linkage analyses. *Am J Hum Genet* 2007; 81:559–575. 17701901 10.1086/519795 PMC1950838

[20] Stephens M Smith NJ Donnelly P A new statistical method for haplotype reconstruction from population data. *Am J Hum Genet* 2001; 68:978–989. 11254454 10.1086/319501 PMC1275651

[21] Agúndez JA Golka K Martínez C Unraveling ambiguous NAT2 genotyping data. *Clin Chem* 2008; 54:1390–1394. 18664443 10.1373/clinchem.2008.105569

[22] Benjamini Y Hochberg Y Controlling the false discovery rate: a practical and powerful approach to multiple testing. *J Roy Statist Soc Ser B* 1995; 57:289–300.

[23] Altman DG Bland JM Diagnostic tests 2: Predictive values. *BMJ* 1994; 309:102. 8038641 10.1136/bmj.309.6947.102 PMC2540558

[24] Baksi SN Hughes MJ Chronic vitamin D deficiency in the weanling rat alters catecholamine metabolism in the cortex. *Brain Res* 1982; 242:387–390. 6288172 10.1016/0006-8993(82)90331-6

[25] Kesby JP Cui X O’Loan J Developmental vitamin D deficiency alters dopamine-mediated behaviors and dopamine transporter function in adult female rats. *Psychopharmacology (Berl)* 2010; 208:159–168. 19921153 10.1007/s00213-009-1717-y

[26] Jiang P Zhang LH Cai HL Neurochemical effects of chronic administration of calcitriol in rats. *Nutrients* 2014; 6:6048–6059. 25533012 10.3390/nu6126048 PMC4277014

[27] Kesby JP Eyles DW Burne TH The effects of vitamin D on brain development and adult brain function. *Mol Cell Endocrinol* 2011; 347:121–127. 21664231 10.1016/j.mce.2011.05.014

[28] Poon AH Gong L Brasch-Andersen C Very important pharmacogene summary for VDR. *Pharmacogenet Genomics* 2012; 22:758–763. 22588316 10.1097/FPC.0b013e328354455c PMC3678550

